# Phytochemistry and Antioxidant Activities of the Rhizome and Radix of *Millettia speciosa* Based on UHPLC-Q-Exactive Orbitrap-MS

**DOI:** 10.3390/molecules27217398

**Published:** 2022-10-31

**Authors:** Jianguang Zhang, Junjun Wang, Yue Wang, Ming Chen, Xuemin Shi, Xiaoping Zhou, Zhifeng Zhang

**Affiliations:** 1 Tibetan Plateau Ethnic Medicinal Resources Protection and Utilization Key Laboratory of National Ethnic Affairs Commission of the People’s Republic of China, Southwest Minzu University, Chengdu 610041, China; 2 Qin Zhou Provincial Health School, Qinzhou 535009, China

**Keywords:** antioxidants, chemical constituent, *Millettia speciosa*, multivariate analysis

## Abstract

The root of *Millettia speciosa* Champ. (MSCP) is used in folk medicine and is popular as a soup ingredient. The root is composed of the rhizome and radix, but only the radix has been used as a food. Thus, it is very important to compare the chemical components and antioxidant activities between the rhizome and radix. The extracts were analyzed by UHPLC-Q-Exactive Orbitrap-MS and multivariate analysis, and the antioxidant activities were evaluated by 2,20-azinobis (3-ethylbenzothiazo-line-6-sulfonic acid) diammonium salt (ABTS) and 2,2-diphenyl-1-picrylhydrazyl (DPPH) radical scavenging assays. Ninety-one compounds were detected simultaneously and temporarily identified. Ten compounds were identified as chemical markers to distinguish the rhizome from the radix. The antioxidant activities of the radix were higher than the rhizome. Correlation analysis showed that uvaol-3-caffeate, 3-O-caffeoyloleanolic acid, and khrinone E were the main active markers for antioxidant activity, which allowed for the rapid differentiation of rhizomes and the radix. Therefore, it could be helpful for future exploration of its material base and bioactive mechanism. In addition, it would be considered to be used as a new method for the quality control of *M. speciosa*.

## 1. Introduction

*Millettia speciosa* Champ. belongs to the Leguminosae family. It is a well-known food and medicine ingredient that is mainly found in Guangxi, Guangdong, and Hainan Provinces of China [1]. MSCP is called Niudali in southern China, which means that it has a strong power, like a bull. The MSCP is commonly used to make soups with pig bones, which can strengthen the functions of the immune system and promote anti-inflammatory and anti-tumor effects. It is also made into various functional products, such as MSCP powder, MSCP tea, and MSCP wine. In previous studies, the chemical constituents of MSCP mainly include polysaccharides, flavonoids, alkaloids, terpenoids, and phenylpropanoids [2,3,4]. Polysaccharides are major bioactive components in the aqueous extracts of MSCP [5,6]. Pharmacological studies showed that the aqueous extracts of MSCP exhibited antifatigue, immunomodulatory, antioxidative, anti-hepatitis, and analgesic activities [3,4,5,6,7].

**Figure 1:**
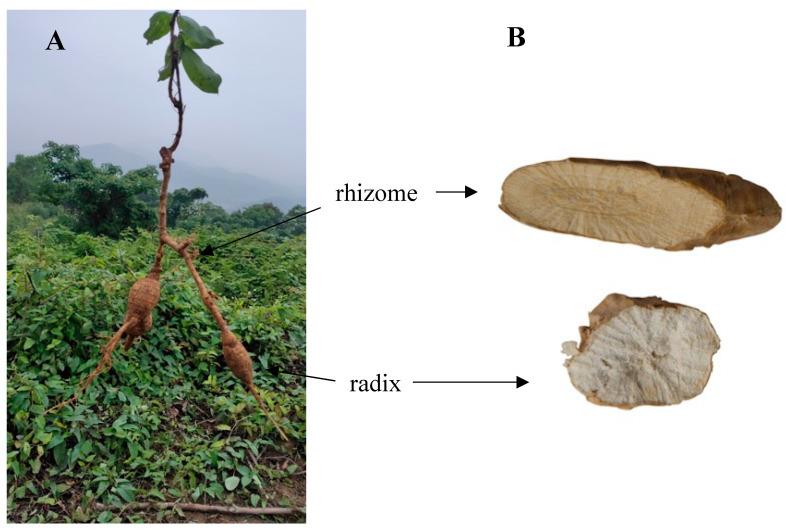
(**A**) An image of the MSCP plant; (**B**) Images of the macroscopic features of the section of MSCP.

However, the root of MSCP consists of the radix and rhizome. Usually, the radix is swollen and powdery, commonly known as Niudali potato, whereas the rhizome is almost lignified and fibrous (Figure 1). Only the radix has been traditionally consumed [8]. Moreover, the price of the radix is higher than that of the rhizome in herbal markets. Therefore, the rhizome is often mixed into the radix before sale, which greatly affects the quality of the radix. Previous studies have mainly focused on the chemical composition and activity of MSCP [1,6,7,9]. A comparative analysis of alcohol soluble components from the rhizome and radix of MSCP showed that the contents of flavonoids, saponins, and alkaloids in the rhizome were greater than those of the radix using ultraviolet–visible spectroscopy [10]. The antioxidant activities of the different parts of MSCP were also compared, and the activity of the root was higher than that of the stem, leaf, and flower [11]. The root of MSCP showed a good antioxidant activity [11]. The root is composed of the rhizome and the radix (Figure 1), but their texture and morphological characteristics are significantly different. There are no reports on the difference in chemical constituents and antioxidant activities between the rhizome and the radix of MSCP.

Ultra-high performance liquid chromatography coupled with the quadrupole time of flight mass spectrometry (UHPLC-QTOF-MS/MS) has been widely used in the separation and structural analysis of complex systems, such as traditional Chinese medicines, due to its rapid separation, high efficiency, high sensitivity, high resolution, and molecular weight accuracy [12]. It can be widely used for the chemical identification of different base sources and different medicinal parts of herbal medicines [13,14,15]. Recently, UHPLC-Q-Exactive Orbitrap MS, with a high selectivity and effectivity, was widely used in the identification of chemical constitutions

In this study, the characteristic chemical components in twenty-eight batches of MSCP were qualitatively analyzed by UHPLC-Q-Exactive Orbitrap-MS. Moreover, principal component analysis (PCA) and orthogonal partial least squares discriminant analysis (OPLS-DA) were used to distinguish the differences between the rhizome and the radix of MSCP. In addition, the antioxidant activities were evaluated by the scavenging rate of ABTS and DPPH. Gray relational analysis (GRA) and partial least squares (PLS) were used to identify potential bioactive markers and to assess the correlation between the characteristic compounds and antioxidant activities.

## 2. Results and Discussion

### 2.1. Optimization of Chromatographic Conditions 

**Figure 2:**
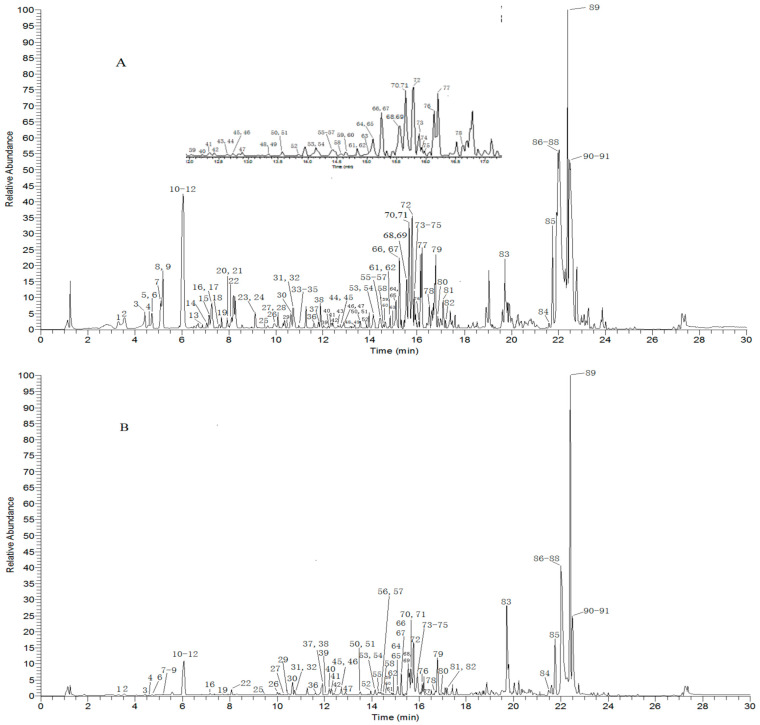
The BPI chromatograms of MSCP rhizome (**A**) and radix (**B**) based on UHPLC-Q-Exactive Orbitrap-MS in negative mode.

In our previous study, we compared the effectiveness of the two columns in the analysis of MSCP samples and found that the ACQUITY HSS T 3 column (100 mm × 2.1 mm, 1.8 μm) was more suitable for the analysis of MSCP because of the greater number of compounds detected and separated. The chromatographic separation was optimized by investigating parameters, such as organic phases (acetonitrile and methanol), with mobile phases (water, water-containing formic acid, and water-containing formic acid and ammonium), and temperatures (20, 25, 30, 35, and 40 °C). The optimal mobile phase consisted of 0.1% aqueous formic acid-acetonitrile with 35 °C, due to a superior peak pattern, better response value, and peak shapes. The base peak ions (BPIs) of rhizome and radix samples obtained in the negative ion mode are shown in Figure 2.

### 2.2. Identification of Components in the Rhizome and Radix Based on UHPLC-Q-Exactive Orbitrap MS

Under the optimal chromatographic and MS conditions, 91 compounds were unambiguously identified, including 71 flavones, 10 phenolic acids, 7 triterpenes, 3 alkaloids, and 10 fatty acid derivatives. Among them, 7 compounds were identified with corresponding reference standards (peaks 10, 28, 40, 55, 70, 72, and 81); however, the remaining 84 compounds were tentatively assigned in accordance with previously published MS data and literature. The retention times, MS data, and fragment ions of all detected compounds are shown in Table 1.

#### 2.2.1. Identification of Flavones, Isoflavones, Flavone Glycosides, and Isoflavone Glycosides

The flavonoid compounds reported in *M. speciosa* were mainly divided into several types, including flavones, isoflavones, flavanones, and chalcones. The fragmentation pathways of flavonoids were followed by Retro-Diels-Alder (RDA) [16]. In this study, a total of 30 flavones, isoflavones, and glycosides were identified. Compounds **37**, **41**, **45**, **60**, **61**, and **65** were isomers that all showed ion peaks at *m/z* 283 [M-H]^−^ and 285 [M+H]^+^ and had a similar molecular formula of C_16_H_12_O_5_. Compound **37** produced fragment ions at *m/z* 270.0515 [M+H-CH_3_]^+^, 242.0564 [M+H-CH_3_-CO]^+^, 225.0535 [M+H-CH_3_-CO-H_2_O]^+^, and 197.0583 [M+H-CH_3_-2CO-H_2_O]^+^, and it yielded RDA fragment ions at *m/z* 137 (^1,3^A^+^) in positive mode, indicating that one hydroxy group was attached to ring A and one hydroxy group and one methoxy group were attached to ring B. Compound **37** was tentatively identified as 5,4′-dihydroxy-3′-methoxy-isoflavone [1]. Compounds **41**, **45**, **60,** and **65** were tentatively identified as isoprunetin, 2′-hydroxyformononetin, calycosin, and glycitein, respectively, based on data reported in the literature [9] and the RDA fragmentation pathway. Similarly, compound **61** was deduced to be 3′,4′-dihydroxy-7-methoxyisoflavone [9].

Compound **19** showed an [M-H]^−^ ion at *m/z* 563.1396 with the molecular formula of C_26_H_28_O_14_, which further produced fragment ions at *m/z* 431.0963 [M-H-132]^−^ and 269.0448 [M-H-132-162]^−^. According to the literature [17,18], compound **19** was tentatively identified as apiin. Compounds **24** and **35** produced an [M-H]^−^ ion at *m/z* 445.1127 and 445.1131, respectively, with the same molecular formula of C_22_H_22_O_10_. Their MS^2^ spectra produced a fragment ion at *m/z* 283, indicating the loss of a hexose moiety. Compound **24** showed fragment ions at *m/z* 268.0370, 240.0420, 224.0464, 212.0472, and 135 (^1,3^A^−^); therefore, it was tentatively identified as calycosin-7-O-beta-D-glucoside [19]. However, compound **35** gave characteristic fragment ions at *m/z* 151 (^1,3^A^−^) and 132 (^1,3^B^−^) by the C-ring RDA fragment, which showed that two hydroxy groups were attached to the A-ring and one methoxy group was attached to the B-ring. Compound **35** was tentatively identified as sissotrin. Similarly, according to the reported literature and MS data in Table 1, compound **31** was tentatively identified as yuankanin [20].

Compound **27** showed an [M+H]^+^ ion at *m/z* 271.0597 (C_15_H_10_O_5_). It produced fragment ions at *m/z* 253.0482 and 137.0230 (^1,3^A^+^) in positive mode. Therefore, compound **27** was tentatively assigned as 5,3′,4′-trihydroxyflavone [1]. Using a similar method, compound **38** was tentatively assigned as daidzein [9]. Compounds **46** and **75** showed an [M-H]^−^ ion peak at *m/z* 297.0398 and 297.0396, respectively, with the same formula of C_16_H_9_O_6_. Compound **46** produced an RDA fragment ion at *m/z* 135 (^1,3^A^−^); thus, compound 46 was tentatively identified as griffonianone H [9]. Compound **75** showed characteristic fragment ions at *m/z* 133 (^1,3^A^−^-H_2_O) and 161 (^0,3^B^−^), indicating that two hydroxy groups were attached to ring A. Thus, compound **75** was tentatively identified as 5,7-dihydroxy-3′,4′-methylenedioxyisoflavone [9].

Compounds **48**, **54**, **58**, **73**, and **74** produced an [M-H]^−^ ion at *m/z* 297.07 with the same formula of C_17_H_14_O_5_. Their MS ^2^ spectra produced fragment ions at *m/z* 282,269, indicating that there were two methoxy groups. Compounds **48**, **54**, **58**, and **73** yielded fragment ions at *m/z* 167 (^1,3^A^−^+H) and 132 (1,3B^−^) by RDA reactions, indicating that one methoxy group and hydroxy group were assumed to attach to ring A and one methoxy group was assumed to attach to ring B. Compared with MS data and literature data [1,9], compounds **48**, **54**, **58**, and **73** were tentatively determined to be 8-O-methylretusin, afrormosin, alfalfa, and 7-O-methylbiochanin A, respectively. Compound **74** generated the RDA fragment ion at *m/z* 135 (^1,3^A^−^), indicating that one hydroxy group was attached to ring A. Compound **74** was tentatively identified as cladactin [9].

Compounds **49** and **72** exhibited a molecular ion at *m/z* 269.0799 [M+H]^+^ with the same formula of C_16_H_12_O_4_. Compound **49** was observed at *m/z* 254.0555, with the loss of a CH_3_ (−15Da). The fragment ions at *m/z* 151.0413 (^1,3^A^+^) and 118.0411 (^1,3^B^+^) were generated by the RDA reaction, which indicated that one methoxy group was on ring A and one hydroxy group was on ring B. Compound **49** was tentatively identified as 4′-hydroxy-7-methoxyisoflavone [1]. Compared with the reference standard, compound **72** was confirmed as formononetin [1,9].

Compounds **52**, **59**, and **62** displayed an [M+H]^+^ ion at *m/z* 301 and a product ion at *m/z* 286, indicating the loss of CH_3_ (−15Da). Compounds **52** and **62** produced RDA fragment ions at *m/z* 153 (^1,3^A^+^) and 134 (^1,3^B^+^-CH_3_) in positive mode, indicating that two hydroxy groups were attached to ring A and hydroxy and methoxy groups were attached to ring B. Compounds **59** and **62** produced a fragment ion at *m/z* 286 [M-H-CH_4_]^−^, which demonstrated that the position of methoxy is ortho to hydroxy. Therefore, compounds **52** and **62** were tentatively assigned as 2′-hydroxybiochanin A [1] and pratensein [9], respectively. Compound **59** exhibited a fragmentation ion at *m/z* 93, with the loss of ring B by the breakage of the C3-C9 bond. This indicated that ring B was substituted by one hydroxy group. Compound **59** was tentatively identified as tectorigenin [9,21].

Compound **63** generated an [M+H]^+^ ion at *m/z* 315.0850, with the formula C_17_H_14_O_6_. It showed characteristic fragments at *m/z* 167.0335 (^1,3^A^+^), 152.0096 (^1,3^A^+^-CH_3_), 148.0514 (^1,3^B^+^), and 133.0228 (^1,3^B^+^-CH_3_), which were generated by the RDA reaction. This suggests that one methoxy and hydroxy group was attached to ring A and ring B, respectively. Compound **63** was tentatively identified as 5,4′-dihydroxy-7,3′-dimethoxyisoflavone [1]. Using a similar method, compounds **64**, **69**, **76**, and **80** were tentatively assigned as 3′,5,6,7-tetrahydroxy-4′-methoxyisoflavone [9], pseudobaptigenin [9], krinone E [22], and tricin [23], respectively. Compound **67** exhibited an [M-H]^−^ ion at *m/z* 313.0346 with a molecular formula of C_16_H_10_O_7_. The characteristic fragment ions were observed at *m/z* 285.0393, 269.1279, and 257.0460, corresponding to the losses of CO (−28 Da), CO_2_ (−44 Da), and 2CO (−56 Da), respectively. It displayed RDA fragment ions at *m/z* 151 (^1,3^A^−^), 161 (^1,3^B^−^), and 109 with the breakage of the C3-C9 bond, indicating that ring A and ring B were simultaneously substituted by two hydroxy groups. Compound **67** was tentatively assigned as luteolal.

#### 2.2.2. Identification of Flavonone, Isoflavonone, and Flavonone Glycosides

As shown in Table 1, a total of 18 compounds were assigned to flavonone, isoflavonone, and flavonone glycosides. Compound **16** showed an [M-H]^−^ ion at *m/z* 581.1500 with the molecular formula C_26_H_30_O_15_, which produced a fragment [M-H]^−^ ion at *m/z* 287.0554 by the loss of the glucosyl moiety (294 Da). The continuous loss of H_2_O (−18 Da) yielded fragment ions at *m/z* 269.0446. It also generated an RDA fragment ion at *m/z* 151 (^1,3^A^−^) in negative mode, meaning that ring A was substituted by two hydroxy groups. Compound **16** was tentatively identified as 5,7,3′,4′-tetrahydroxyflavanone-7-alpha-L-arabinofuranosyl-(1->6)-glucoside. Using a similar method, compound **17** was tentatively assigned as eriodictyol-7-O-glucoside [24].

Compounds **23**, **25**, **32**, **53**, and **55** produced an [M-H]^−^ ion at *m/z* 271 with the same formula, C_15_H_12_O_5_. Compounds **23**, **25**, and **32** generated fragment ions at *m/z* 243 [M-H-CO]^−^ and 227 [M-H-CO_2_]^−^. Compounds **23**, **25**, and **32** yielded characteristic fragmentation ions at *m/z* 135 (^1,3^A^−^) and 109 with the breakage of the C_3_-C_9_ bond by RDA reactions, indicating that ring A had one hydroxy group and that ring B had two hydroxy groups. Compounds **23**, **25**, and **32** were tentatively identified as 3′,4′,7-trihydroxyflavanone [25], 3′,4′,7-trihydroxy-isoflavanone, and 2′,4′,7-trihydroxyisoflavanone through a comparison with MS data and literature data. Compound **53** generated fragment ions at *m/z* 253 [M-H-H_2_O]^−^ and 135 (^1,3^A^−^) and was identified as garbanzol. Compound **55** showed fragment ions at *m/z* 151 (^1,3^A^−^) and 119 (^1,3^B^−^), which were definitively identified as naringenin [9] via a comparison with the reference standard.

Compounds **36**, **42**, **44**, and **66** produced an [M-H]^−^ ion at *m/z* 285 with the same formula of C_16_H_14_O_5_, which produced a fragment [M-H]^−^ ion at *m/z* 270 by the loss of CH_3_ (15 Da). Compounds **36** and **44** generated an RDA fragment ion at *m/z* 135 (^1,3^A^−^) in negative ion mode, indicating that ring A had one hydroxy group. Compound **44** exhibited a fragment [M-H]^−^ ion at *m/z* 269 by the loss of CH_4_ (16 Da), meaning that the position of methoxy is ortho to hydroxyl in ring B. Therefore, compounds **36** and **44** were assigned as vestitone [9] and 3′,7-dihydroxy-4′-methoxyisoflavanone, respectively. Compounds **42** and 66 generated fragment ions at *m/z* 149 (^1,3^A^−^) and 135 (^1,3^A-CH_3_) by the RDA reaction, meaning that ring A had one methoxy group. Compounds **42** and **66** were assigned as 3′,4′-dihydroxy-7-methoxyflavanone and 3′,4′-dihydroxy-7-methoxyisoflavanone [9]. Compound **56** produced an [M+H]+ ion at *m/z* 287.0912 with the formula of C_16_H_14_O_5_. In addition, MS^2^ fragment ions at *m/z* 269.0803 [M+H-H_2_O]^+^and 153.0544 (^1,3^A^+^) were generated, meaning that two hydroxy groups were in the ortho-position of ring A, and the structure was tentatively assigned as 6,7-dihydroxy-4′-methoxyisoflavanone.

Compounds **39**, **40**, and **51** yielded an [M-H]^−^ ion at *m/z* 255 with the same formula of C_15_H_12_O_4_. They produced characteristic fragment ions at *m/z* 135 (^1,3^A^−^) and 119 (^1,3^B^−^), indicating that one hydroxy group was attached to ring A and ring B, respectively. Compound **40** was unambiguously identified as liquiritigenin [9] through a comparison with the reference standard. Compounds **39** and **51** were tentatively assigned as dihydrodaidzein [9] and 5,4′-dihydroxyflavanone, respectively. Compounds **43** and **82** produced an [M-H]^−^ ion at *m/z* 315 with the formula of C_17_H_16_O_6_. Compound **43** further yielded a fragment [M-H]^−^ ion at *m/z* 135.0071 (^1,3^A^−^), and it was tentatively assigned as violanone [9]. Compound **82** generated fragment [M-H]^−^ ions at *m/z* 300.0629, 285.0401, and 151.0023 (^1,3^A^−^). Thus, it was tentatively assigned as homoferreirin. Similarly, compound **68** was tentatively assigned as ferreirin [1].

#### 2.2.3. Identification of Chalcone, Dihydrochalcone, and Flavanones

Chalcone, dihydrochalcone, and flavanones give weak RDA fragments and commonly lose ring B by breaking at different positions [16]. Compound **29** exhibited an [M-H]^−^ ion at *m/z* 271.0606 with the formula C_15_H_12_O_5_. It gave characteristic fragment ions at *m/z* 253.050 and 135.043. Compound **29** was tentatively assigned as butein. Compound **34** produced an [M-H]^−^ ion at *m/z* 273.0758, which was 2 Da more than that of compound **29**, indicating a dihydrochalcone. Further fragmentation led to ions at *m/z* 255.0658 [M-H-H_2_O], 167.0336, and 137.0029, indicating that ring B was substituted by one hydroxy group and ring A was substituted by two hydroxy groups. Ring C was also substituted by one hydroxy group. Compound **34** was tentatively identified as 2′,4,4′, α-tetrahydroxydihydrochalcone [25]. Compounds **57** and **78** showed an [M+H]^+^ ion at *m/z* 271 with the same formula of C_16_H_14_O_4_. Compound **57** yielded characteristic fragment [M+H]^+^ ions at *m/z* 151.0386 and 119.0489, indicating that ring B had one hydroxy group. Compound **57** was tentatively assigned as 2′,4-dihydroxy-4′-methoxychalcone [7]. Compound **78** generated a characteristic fragment [M+H]+ ion at *m/z* 123.0438, and it was tentatively assigned as echinatin [1]. Compound **70** yielded an [M+H]+ ion at *m/z* 257.0802 with product ions at 137.0229 and 119.049. Compound **70** was confirmed as isoliquiritigenin [9] with the corresponding reference standard. Compound **13** exhibited an [M-H]^−^ ion at *m/z* 577.1352 (C_30_H_26_O_12_) and produced ions at *m/z* 289.0711 and 125.0299. Compound **13** was tentatively identified as procyanidin B2 [26]. Compound **15** produced an [M-H]^−^ ion at *m/z* 289.0708 with the formula C_15_H_14_O_6_. The characteristic fragment ions at *m/z* 245.0809, 137.0229, 125.0229, and 109.0280 were generated from (-)-epicatechin [26].

#### 2.2.4. Identification of Pterocarpans and Other Flavonoids

**Figure 3:**
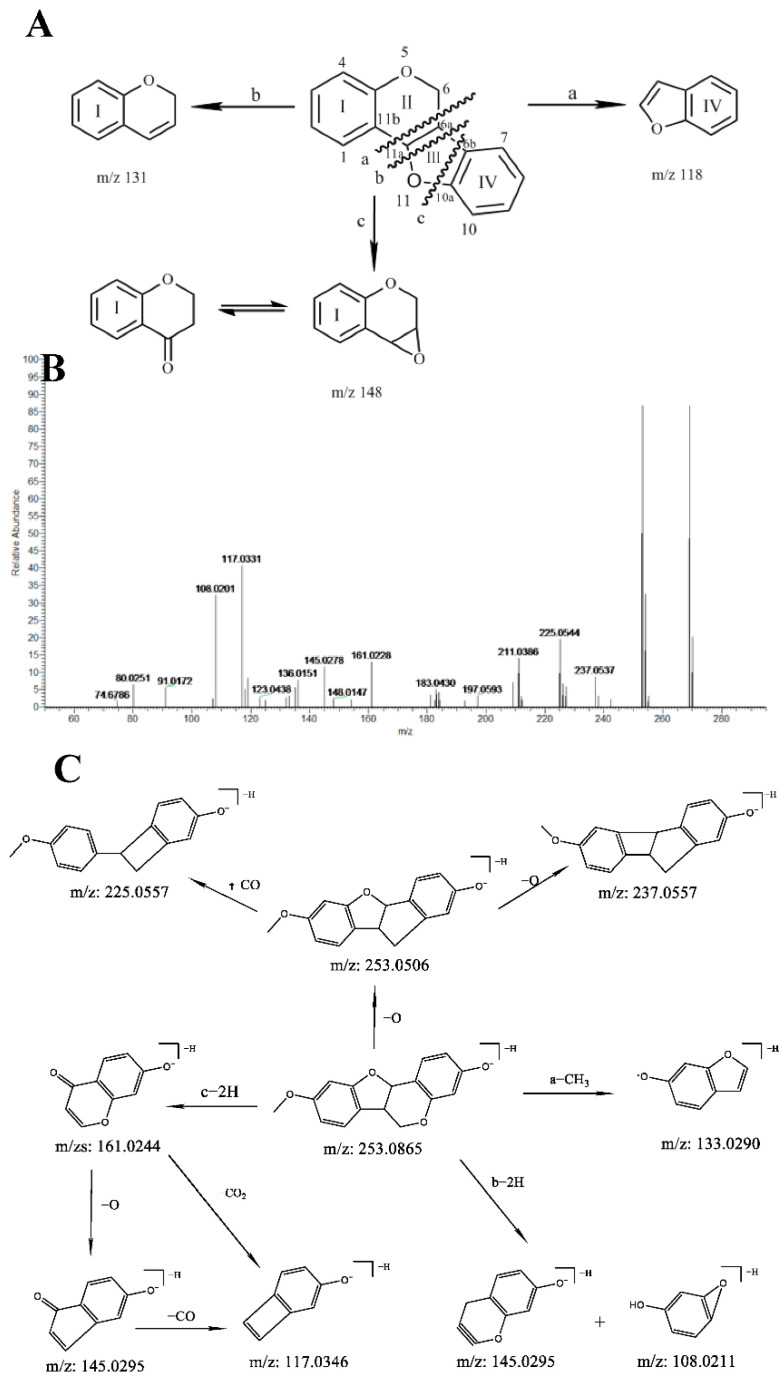
(**A**) Fragmentation pathway of Pterocarpans compounds in MSCP; (**B**) The MS/MS spectrum of compound **50**; (**C**) The hypothesized fragmentation pathway of compound **50**.

Usually, pterocarpan cannot produce RDA reactions because of its tight structure. The proposed fragmentation pathways of pterocarpan compounds are summarized in Figure 3A. The cleavage of the C-C bonds of pterocarpan compounds mainly occurred on the side of C_6a_ and C_11a_ to generate ion fragments a, b, and c. For example, compound **50** displayed [M-H]^−^ ions at *m/z* 269.0812 with the formula C_16_H_14_O_4_. The characteristic fragment in negative mode included 253.0499 [M-H-O]^−^, 237.0547 [M-H-CH_3_-O]^−^, 225.0549 [M-H-CH_3_-CO]^−^, 161.0225 (C_9_H_5_O_3_, c-2H), 145.0278 (C_9_H_5_O_2_, b-2H), 133.0188 (C_8_H_5_O_2_, a-CH_3_), and 117.0331 (C_8_H_5_O, c-2H-CO_2_) in its MS^2^ spectrum (Figure 3B), indicating that ring I was substituted by one hydroxy group and ring IV was substituted by one methoxy group. The hypothesized fragmentation pattern of compound **50** is demonstrated in Figure 3C. Thus, compound **50** was tentatively identified as medicarpin.

Compound **47** produced an [M-H]^−^ ion at *m/z* 285.0759 with the formula C_16_H_14_O_5_. and produced characteristic ions at *m/z* 270.0520 [M-H-CH_3_]^−^, 269.0431 [M-H-CH_4_]^−^, 163.0365 (C_9_H_7_O_3_, a), 148.0190 (C_8_H_4_O_3_, a-CH_3_), and 147.0433 (C_9_H_7_O_2_, b). This indicates that ring I was substituted by one hydroxy group and ring IV was substituted by hydroxy ortho with a methoxy group. Thus, compound **47** was tentatively confirmed as 3,8-dihydroxy-9-methoxypterocarpan. Compounds **77** and **81** showed ions at *m/z* 285 [M+H]^+^ and 283 [M-H]^−^ with the same formula, C_16_H_14_O_4_. Compound **77** exhibited characteristic ions *m/z* 270.0910 [M+H-CH_3_]^+^, 255.0642 [M+H-2CH_3_]^+^, 163.0388 (C_9_H_7_O_3_, b), and 147.0432 (C_8_H_4_O_3_, b-CH_3_) in positive mode, and it was tentatively assigned as homopterocarpin. Compound **81** yielded an [M-H]^−^ ion at *m/z* 268.0367 [M-H-CH_3_]^−^, 239.0341 [M-H-CO_2_]^−^, 223.0393 [M-H-CO_2_-O]^−^, 211.0390 [M-H-CO_2_-CO]^−^, and 195.0451 [M-H-2CO_2_]^−^, but it also produced major product ions at *m/z* 163.0348 (C_9_H_7_O_3_, b) and 151.0385 (C_8_H_7_O_3_, b-C) in positive mode. Compound **81** was confirmed as a mixture using the corresponding reference standards.

**Figure 4:**
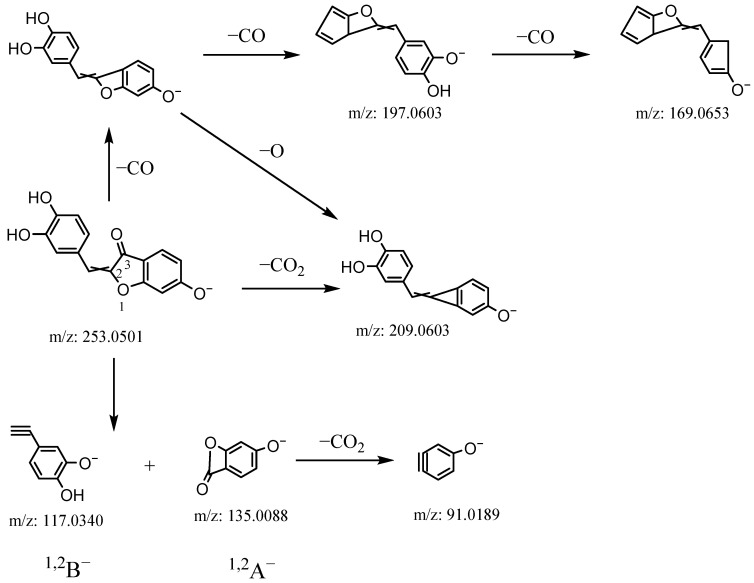
The hypothesized fragmentation pathway of compound **33**.

Compound **33** produced an [M-H]^−^ ion at *m/z* 269.0448 with the formula C_16_H_10_O_5_. In the MS^2^ spectra, it gave ions at *m/z* 241.0495 [M-H-CO]^−^, 225.0524 [M-H-CO_2_]^−^, 213.0547 [M-H-2CO]^−^, 185.0593 [M-H-3CO]^−^, 135.0073 (^1,2^A^−^), 133.0280 (^1,2^B^−^), and 91.0173 (^1,2^A^−^-CO_2_). The hypothesized fragmentation pattern of compound **33** is presented in Figure 4. Thus, compound **33** was tentatively identified as sulfuretin, belonging to aurone.

#### 2.2.5. Identification of Steroid Saponins

Steroid saponins are major biological compounds present in MSCP. The ions of saponins tended to be present in the negative mode, with successive losses of several sugar moieties. In this work, seven steroid saponins were rapidly identified using UHPLC-Q-Exactive Orbitrap MS by matching the structural data combined with the literature data of published compounds. For example, compound **79** yielded an [M-H]^−^ ion at *m/z* 941.5099 with the formula C_48_H_78_O_18_. It gave fragment ions at *m/z* 795, 633, and 457 by the successive losses of deoxyhexose, hexose, and glucuronic acid moieties, respectively. It was tentatively confirmed as soyasaponin I. Compound **85** generated an [M+H]^+^ ion at *m/z* 457.3676, and it produced fragment ions at *m/z* 439.3570 [M+H-H_2_O]^+^, 381.3133 [M+H-2H_2_O]^+^, 248.1692, and 191.1788. It was tentatively identified as betulinic acid. Compounds **86**, **87**, and **88** showed an [M-H]^−^ ion at *m/z* 617 with the same formula, C_39_H_54_O_6_. Compound **86** exhibited fragment ions at *m/z* 453, 163, 145, and 119 and was tentatively identified as 27-p-coumaroyloxyursolic acid. Compounds **87** and **88** gave fragment ions at *m/z* 437, 179, 161, and 134, respectively. They were tentatively assigned as 3-β-O-trans-caffeoyl-betulinic acid and 3-O-caffeoyl-oleanolic acid, respectively. Compounds **90** and **91** gave an [M-H]^−^ ion at *m/z* 603 with the same formula, C_39_H_56_O_5_. They gave fragment ions at *m/z* 179, 161, and 134. Compounds **90** and **91** were tentatively assigned as betulin-3-caffeate and uvaol-3-caffeate, respectively.

#### 2.2.6. Identification of Alkaloids

Alkaloid compounds tend to produce signals in negative ion mode. In this work, three alkaloid compounds were rapidly identified. Compounds **10** and **12** showed an [M+H]^+^ ion at *m/z* 188 with the same formula, C_11_H_9_O_2_N. The fragment ions at *m/z* 170 [M+H-H_2_O]^+^, 143 [M+H-COOH]^+^, 118 [M+H-C_2_H_2_COOH]^+^, and 91 [M+H-C_4_H_5_COOH]^+^ were produced in the MS^2^ spectrum. They were tentatively identified as trans-3-indoleacrylic acid and indole-3-acrylic acid, respectively. Compound **11** generated an [M+H]^+^ ion at *m/z* 247.1434, and it further produced fragment ions at *m/z* 188.0701 [M+H-NCH3]^+^ and 146.0598 [M+H-NCH_3_-CO_2_]^+^. It was unambiguously identified as hypaphorine using the reference standard.

#### 2.2.7. Identification of Phenolic Acids and Their Derivatives

Phenolic acids and derivatives extensively exist in the plant. Deprotonated ions were detected in negative ion mode. Compounds **1**, **2**, and **6** produced an [M-H]^−^ ion at *m/z* 315 with the same formula, C_13_H_16_O_9_. As shown in Table 1, they produced characteristic fragment ions at *m/z* 152 [M-H-glu]^−^ and 108 [M-H-glu-CO_2_]^−^ by the successive elimination of Glu and CO_2_ groups, respectively. They were tentatively identified as protocatechuic acid-4-glucoside, protocatechuic acid-3-glucoside, and protocatechuic acid-2-glucosid. Compounds **4** and **5** showed an [M-H]^−^ ion at *m/z* 461 with the same formula, C_19_H_26_O_13_. In the MS^2^ spectrum, the successive elimination of pentose, hexose, CH_3_, and CO_2_ groups generated fragment ions at *m/z* 329, 167, 152, and 108, respectively. Compounds **4** and **5** were tentatively assigned as saccharumoside C and saccharumoside D, respectively. Compounds **7** and **8** generated an [M-H]^−^ ion at *m/z* 447 with the same formula, C_18_H_24_O_13_. In the MS^2^ spectrum, the successive elimination of pentose, hexose, and CO_2_ groups generated fragment ions at *m/z* 315, 152, and 108, respectively. Compounds **7** and **8** were tentatively assigned as 5-{[2-O-(beta-d-apiofuranosyl)-beta-d-glucopyranosyl]oxy}-2-hydroxybenzoic acid and 4-hydroxy-5-(3′,4′,5′-trihydroxyphenyl)-valeric acid-O-methyl-O-glucuronide, respectively. By using a similar approach, compounds **20**, **21**, **22**, and **26** were tentatively assigned as seguinoside K [27], albibrissinoside B [27], apiosylglucosyl-4-hydroxybenzoate, and khaephuoside B [27] through a comparison of the structural data and literature data. Compound **28** produced an [M-H]^−^ ion at *m/z* 137.0229, which further yielded a fragment ion at *m/z* 93.0329 by the loss of the CO_2_ group. It was identified as salicylic acid [9] using the reference standard.

#### 2.2.8. Identification of Phenols, Fatty Acid and Other Compounds

Compounds **9** and **14** exhibited an [M-H]^−^ ion at *m/z* 477 with the same formula of C_20_H_30_O_13_. Their MS^2^ spectrum showed fragment ions at *m/z* 345, 183, 168, and 153, corresponding to the loss of pentose, hexose, and two CH_3_ molecules. Compounds **9** and **14** were tentatively assigned as shamiminol and kelampayoside A, respectively. Using a similar method, compound **18** was tentatively assigned as diosbulbinoside D.

Compound **30** exhibited an [M-H]^−^ ion at *m/z* 187.0963 with the formula C_9_H1_6_O_4_. The fragment ions at 169.0850 [M-H-H_2_O]^−^, 143.1064 [M-H-CO_2_]^−^, and 125.0956 [M-H-CO_2_-H_2_O]^−^ were observed in the MS^2^ spectrum. It was identified as a fatty acid in *M. speciosa* and tentatively assigned as azelaic acid [9]. Through a similar method, compounds **71**, **83**, and **84** were tentatively assigned as 9,12,13-trihydroxy-10-octadecenoic acid [1], 9-hydroxyoctadeca-10,12-dienoic acid [1], and 2-pentadecanone [1], respectively. Compound **3** showed an [M+H]^+^ ion at *m/z* 421.1330 with the formula C_17_H_24_O_12_. It gave fragment ions at *m/z* 289 and 127, corresponding to the loss of pentose and hexose, respectively. It was tentatively identified as NCGC00380493-01. Compound **89** produced an [M-H]^−^ ion at *m/z* 339.2321 with the formula C_23_H_32_O_2_. The fragment ion at *m/z* 163 was obtained, and compound **89** was tentatively assigned as 2,2′-methylenebis.

### 2.3. Discrimination of Chemical Profiles of Rhizome and Radix

As shown in Figure 2, a total of 91 chemical constituents were identified in the rhizome and radix samples. It is worth noting that all of the intensities of the chemical constituent peaks of the rhizome were stronger than those of the radix under the same analytical conditions. In total, the intensities of the peaks were comparatively low, within 6.2–8, 8.5–10, and 13.5–14 min in the radix, and the intensities of peaks 5, 9, 13–15, 17, 18, 20, 21, 23, 24, 28, 33–35, 43, 44, 48, and 49 were almost undetectable in the radix samples. To establish representative chromatographic fingerprints, the established method was applied to analyze 16 batches of rhizome and 12 batches of radix. There were 91 common compounds identified in the MS spectra fingerprints from the rhizome and radix. However, considering many common peaks and a large amount of peak area data, it was difficult to distinguish rhizome and radix samples using this information. Multivariate analysis, including PCA and OPLS-DA, was performed on mass spectral data sets using SIMCA-P14.1 software. The loading plot from OPLS-DA together with the variable importance in the projection (VIP) were applied to reveal potential markers [14,15,16]. Thus, it is necessary to use multivariate analysis to reduce the dimensionality of the primal data.

### 2.4. Principal Components Analysis (PCA)

**Figure 5:**
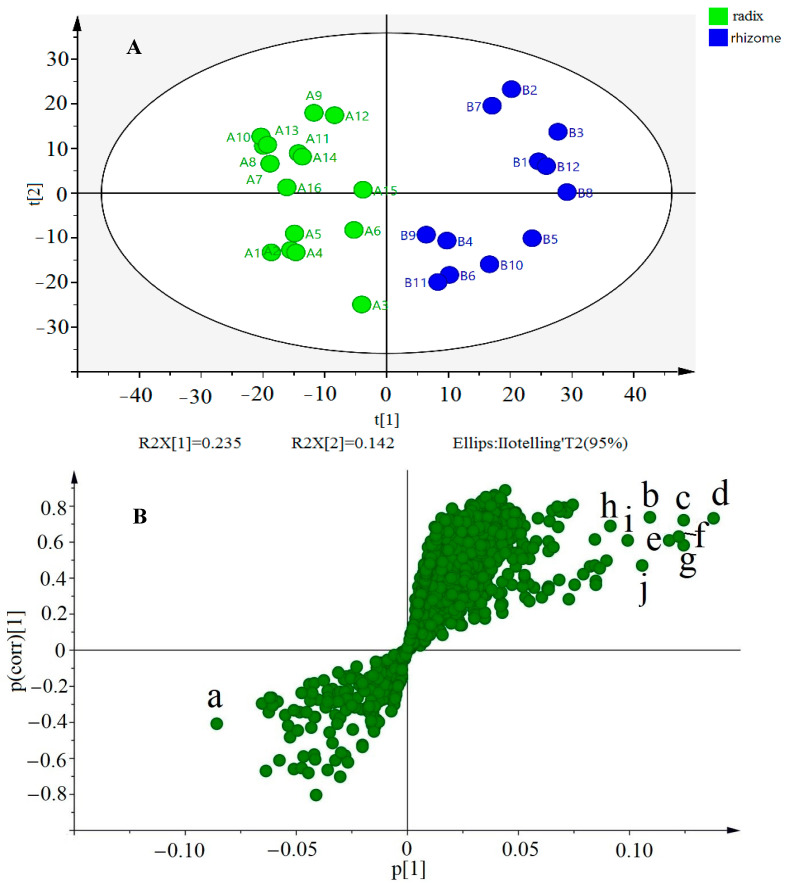
(**A**) The PCA scores plot of rhizome and radix; (**B**) The S plot of the rhizome and radix.

To efficiently visualize the differences between 16 batches of rhizomes and 12 batches of radix from MSCP, PCA was applied to analyze the MS spectral data using SIMCA-P14.1 software. The parameters R^2^ (cum) and Q^2^ (cum) are generally used to explain the quality and reliability of the models. The PCA results are presented in two score plots (Figure 5A), and 28 batches of MSCP samples were divided into two types containing rhizome or radix (R^2^ (cum) = 0.235 and Q^2^ (cum) = 0.377). The samples from different parts were clearly distinguished into two groups according to the PCA model. As shown in Figure 5A, the radix (A1–A16) of MSCP is displayed on the left side of the score plot, whereas rhizome (B1–A12) is displayed on the right side of the plot, indicating a difference between the rhizome and radix in terms of chemical composition. Therefore, it is possible to distinguish the rhizome and radix samples based on UHPLC-Q-Exactive Orbitrap MS fingerprint analysis with PCA. These results revealed the distinctive differences in chemical composition between the rhizome and radix samples of MSCP.

### 2.5. Chemical Markers to Distinguish the Rhizome from Radix with OPLS-DA

To identify chemical markers unique to the rhizome and radix samples, OPLS-DA was utilized to further process the MS spectral data by SIMCA-P14.1 software. The loading S-plot was used to characterize the chemical difference between the rhizome and the radix. In Figure 5B, the spots located at the end of the plot indicate that the contribution of the variable to the differentiation is higher. In this S-plot, every spot represents an ion t_R_-m/z pair. The *x*-axis presents the alterable contributions of the variables, whereas the *y*-axis presents the alterable confidence levels of the variables. Therefore, when the distance between the ion t_R_-m/z pair spots and zero increases, the confidence level of the difference between the rhizome and radix also increases. Thus, those spots located at the end of the plot were tentatively regarded as potential chemical markers, leading to differences between the rhizome and radix samples.

In addition, the VIP value was employed to confirm the potential markers, which represent the differentiation between the rhizome and radix. When the VIP is higher, the variables are more important to the model. The S-plot, together with the variables of VIP ≥1, suggests the influence of the differentiation of samples using OPLS-DA.

From Figure 5B, one ion (a) in the bottom left corner and eight ions (b–j) in the top right corner of the S-plot contributed the most to the differentiation of the rhizome and radix. Combining this analysis with the base peak ion (BPI) chromatogram, ten compounds generated from peak 79 (soyasaponin I, VIP 2.66), peak 89 (2,2′-methylenebi, VIP 3.29), peak 90 (betulin-3-caffeate, VIP 4.42), peak 91 (uvaol-3-caffeate, VIP 3.91), peak 88 (3-*O*-caffeoyloleanolic acid, VIP 3.90), peak 76 (khrinone E, VIP 3.83), peak 87 (3-β-*O*-*trans*-caffeoyl betulinic acid, VIP 4.25), peak 82 (homopterocarpin, VIP 2.78), peak 85 (betulinic acid, VIP 3.20), and peak 86 (27-p-coumaroyloxyursolic acid, VIP 3.95) were confirmed to be the most characteristic compounds. Therefore, these 10 chemical constituents were considered as potential markers to distinguish the rhizome and radix of MSCP samples, and this method could be applied for the differentiation of the rhizome and radix in other samples.

### 2.6. Antioxidant Activity Test Results

The UHPLC-Q-Exactive Orbitrap MS results show that MSCP extracts mainly contained flavonoids, phenolic acids, and steroid saponins. Previous studies have confirmed that plants with antioxidant activity are closely related to their chemical compounds, such as flavonoids, phenolic acids, and steroid saponins [28]. Antioxidants are closely associated with human health. The damaging effects of free radicals can directly or indirectly lead to diseases and cancers [29]. The antioxidant activities of different extracts between the rhizome and radix of MSCP were determined by ABTS and DPPH antioxidant assays. As shown in Table 2, different batches of MSCP showed different antioxidant abilities, compared with the IC_50_ values. In addition, a significant difference in the IC_50_ values between rhizomes and radixes was observed using ABTS and DPPH antioxidant tests. The IC_50_ values of the rhizome and radix extracts were 5.05–10.13 μg/mL and 2.09–4.56 μg/mL in the ABTS test, respectively. The IC_50_ values of the rhizome and radix extracts were 5.86–11.86 μg/mL and 2.76–5.77 μg/mL in the DPPH test, respectively. The lower IC_50_ values indicate a higher antioxidant activity [30]. Therefore, in both the ABTS and DPPH antioxidant assays, the antioxidant activity of radix samples was obviously higher than that of rhizome samples. The results indicate that both rhizome and radix extracts of MSCP presented remarkable antioxidant activities in vitro. However, it is still unknown whether the variability in the chemical components is related to differences in antioxidant activity and whether the variability influences the ability to discriminate between the rhizomes and radix. Therefore, further study is necessary to reveal the relationship between chemical components and antioxidant efficacy via statistical analysis.

### 2.7. Correlations between the Characteristic Chemical Compounds and Antioxidant Activities

#### 2.7.1. GRA

As shown in Table 3, the GRA results indicate that the relational grade between the 10 peaks and the antioxidant activity was in the range of 0.5661–0.7846. In ABTS assays, the relational degrees of peaks a, c, d, e, and f exceeded 0.7000, which indicates that these compounds play important roles in the correlation. In DPPH assays, the relational grade of peaks b, c, d, e, and f were more than 0.7000, and these peaks may be closely related to DPPH antioxidant activity. Therefore, betulin-3-caffeate (peak 90), uvaol-3-caffeate (peak 91), 3-O-caffeoyloleanolic acid (peak 88), and khrinone E (peak 76) were assigned as essential markers for antioxidant activity, and the contents of these components may be related to antioxidant activity. Notably, the common peak c had the highest relational grade in both ABTS and DPPH assays. Thus, it is likely to be the most relevant compound to reflect antioxidant activity. To determine whether the gray correlation degree was positively or negatively correlated with biological activity, the PLS model was used for further statistical analysis, as described below [31].

#### 2.7.2. PLS Model Analysis

**Figure 6:**
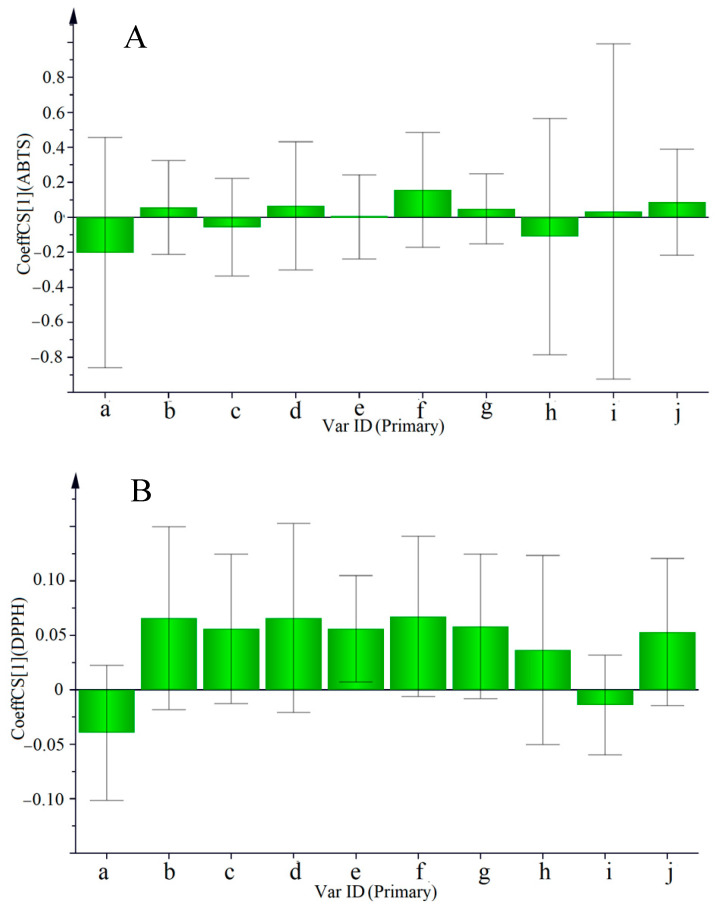
Regression coefficients of antioxidant activity obtained with PLS (**A**) is the regression coefficient figure of ABTS antioxidant activity; (**B**) is the regression coefficient figure of DPPH antioxidant activity).

PLS analysis was further used to predict the correlation between the characteristic chemical components and antioxidant activity. Figure 6 was drawn to reflect the correlation between 10 peaks and antioxidant activity. The regression equations obtained by the PLS model are as follows:

Y_1_(ABTS) = −0.202X_a_ + 0.056X_b_ − 0.056X_c_ + 0.066X_d_ + 0.003X_e_ + 0.157X_f_ + 0.048X_g_ − 0.110X_h_ + 0.033X_i_ + 0.086X_j_ (1)

Y_2_(DPPH) = −0.039X_a_ + 0.065X_b_ + 0.056X_c_ + 0.066X_d_ + 0.056X_e_ + 0.067X_f_ + 0.058X_g_ + 0.037X_h_ − 0.014X_i_ + 0.053X_j_ (2)

As shown in Figure 6A and Equation (1), the areas of peaks b, d, e, f, g, i, and j showed a clear positive correlation with ABTS antioxidant activity, whereas peaks a, c, and h were negatively correlated with antioxidant activity. Figure 6B and Equation (2) show that the areas of peaks b, c, d, e, f, g, h, and j are positively correlated with DPPH antioxidant activity, whereas peaks a and i are negatively correlated with antioxidant activity. Therefore, 2,2′-methylenebis (peak 89), uvaol-3-caffeate (peak 91), 3-O-caffeoyloleanolic acid (peak 88), khrinone E (peak 76), 3-β-O-trans-caffeoyl betulinic acid (peak 87), and 27-p-coumaroyloxyursolic acid (peak 86) represent compounds that significantly contribute to the pharmacological effects of MSCP. Khrinone E (peak 76) showed the highest regression coefficient and was considered to have the highest contribution to antioxidant activity.

Therefore, correlation analysis shows that uvaol-3-caffeate (peak 91), 3-O-caffeoyloleanolic acid (peak 88), and khrinone E (peak 76) are the main active markers for the antioxidant activity of MSCP.

## 3. Materials and Methods

### 3.1. Reagents and Chemicals

HPLC-grade acetonitrile and methanol were obtained from Fisher Scientific Co. (Loughborough, UK). Deionized water was used. High-purity (>98.0%) hypaphorine, salicylic acid, liquiritigenin, naringin, isoliquiritigenin, formononetin, and maackiain were purchased from the National Institutes for Food and Drug Control (Beijing, China). All other chemicals were of analytical grade. The ABTS and DPPH were purchased from Sigma–Aldrich (St. Louis, MO, USA).

### 3.2. Sample Collection

The MSCP was collected from different locations in Guangxi Province and authenticated by Professor Zhifeng Zhang (Institute of Qinghai-Tibetan Plateau, Southwest Minzu University). The sample information is shown in Table 2. The fresh sample was sectioned and dried in the sun. The samples were kept in the herbarium of Qin Zhou Provincial Health School (Qinzhou, China).

### 3.3. Preparation of Standard and Sample Solutions

Standard stock solutions of hypaphorine, salicylic acid, liquiritigenin, naringin, isoliquiritigenin, formononetin, and maackiain were prepared in methanol/water (50% *v*/*v*), and the final concentration was 0.1 mg/mL. The stock solutions were further diluted and stored in a refrigerator at −20 °C until UHPLC-Q-Exactive Orbitrap MS analysis.

The dried sample was ground to a powder and saved in desiccators at normal temperature for future use. Subsequently, the sample powder (0.3 g) was extracted with 10 mL of 70% methanol in an ultrasonic water bath for 30 min at room temperature. The solution was filtered through a 0.22 μm microfiltration membrane.

### 3.4. Instrumentation and Chromatographic Conditions

The solutions were measured using a Thermo Scientific™ Vanquish™ Flex UHPLC (Thermo Fisher Scientific Inc., Waltham, MA, USA) equipped with a binary solvent system, autosampler, and full UV wavelength spectrophotometer. The chromatographic conditions were set and modified according to our previous work [32]. The chromatographic separation was performed with an ACQUITY HSS T 3 column (100 mm × 2.1 mm, 1.8 μm). The solvent system consisted of 0.1% aqueous formic acid (A) and acetonitrile (B), with the following gradient elution program: 3–3% B (0–1.5 min), 3–15% B (1.5–5 min), 15–30% B (5–12 min), 30–95% B (12–22 min), 95–95% B (22–25 min), 95–3% B (25–26 min), and 3–3% B (26–30 min). The flow rate was 0.3 mL/min. The column was kept at 35 °C, and the injection volume was 3 μL.

Mass spectrometry was performed on a Thermo UHPLC-Q-Exactive Orbitrap mass spectrometer equipped with an electrospray ionization (ESI) source. Both positive and negative ionization modes were applied to acquire a scanning range from 100 to 1000 Da with a scanning time of 0.2 s and a 30 min detection period. The MS parameters were set as follows: the capillary voltage was set to 3.5 kV (positive mode) and 3.2 kV (negative mode); the source and desolvation temperatures were 100 and 350 °C, respectively; the drying gas flow rate was 600 L/h; and the cone flow rate was 50 L/h. Finally, processing and analysis of the data were carried out using Xcalibur 2.1 software (Thermo Fisher Scientific, Bremen, Germany).

### 3.5. Determination of Antioxidant Activity

#### 3.5.1. ABTS Activity Assay

The ABTS assay was performed according to a previously described study by Wang et al. [11] with a few modifications. The ABTS aqueous solution (7 mM) was mixed with K_2_S_2_O_8_ (2.45 mM) and protected from light at room temperature for 12 h. The configured ABTS^+^ solution was diluted with anhydrous ethanol, and an absorbance of 0.70 ± 0.02 was measured at 734 nm for ABTS^+^ analysis. Five different concentrations of sample solutions were prepared by diluting with anhydrous ethanol. Then, 0.4 mL of the sample extract was mixed with 4 mL of the diluted ABTS^+^ solution at 25 °C for exactly 5 min. The absorbance was determined at 734 nm. The ABTS activity was calculated using the following Equation.

ABTS activity (%) = (1 − A_sample_/A_blank_) × 100% (3)

where A_sample_ is the absorbance of 0.4 mL of sample extract combined with 4 mL of the diluted ABTS^+^ solution, and A_blank_ is 0.4 mL of anhydrous ethanol combined with 4 mL of the diluted ABTS^+^ solution. The half inhibition concentration (IC_50_) value was evaluated using a regression equation from serial concentrations of the sample extract.

#### 3.5.2. DPPH Activity Assay

The DPPH scavenging capacity was determined as previously described by Wang et al. [11] with a small modification. The extracts were concentrated to five different concentrations by distillation to prepare the sample solutions. A 4 mL DPPH (0.04 mg/mL) solution was mixed with 1 mL of the sample and kept in the dark at 25 °C for 30 min. The absorbance was recorded at 517 nm, and DPPH activity was calculated via the following Equation.

DPPH activity (%) = (1 − A_sample_/A_blank_) × 100% (4)

where A_sample_ is the absorbance of 1 mL of sample extract combined with 4 mL of the diluted DPPH solution, and A_blank_ is 1 mL of anhydrous ethanol combined with 4 mL of the diluted DPPH solution. Regression analysis of the data was used to estimate the IC_50_ values.

### 3.6. Statistical Analysis

The processed data with accurate mass were exported from Xcalibur 2.1 software. The match factor and retention time window of the peaks were set to 0.3 ppm and 0.05 min, respectively.

The spectral information was imported into SIMCA software (SIMCA-P 14.1, Umetrics Inc., Umea, Sweden) for multivariate statistical analysis. Principal component analysis (PCA) and orthogonal partial least-squares discriminate analysis (OPLS-DA) were used to distinguish between rhizome and radix samples. The S-plot and the importance in the projection (VIP) were used for predicting potentially characteristic chemical compounds between the rhizome and radix. Molecular formulas were determined using fragment information obtained from the workstation, as well as the established database and information from the literature. The cleavage process of each compound was predicted based on the MS and MS/MS information. Data were compared by one-way ANOVA followed by Turkey’s test for multiple comparisons. A value of *p* < 0.05 was considered to represent a significant difference.

### 3.7. Correlation Analysis

#### 3.7.1. Gray Relational Analysis

GRA was performed using the DPS software (DPS 9.5, China) to calculate the correlation degree between the characteristic chemical compound peak area of MSCP and the antioxidant activities (ABST and DPPH radical scavenging activity). The gray relational grade was calculated with a distinguishing coefficient of 0.5.

#### 3.7.2. Partial Least Squares Analysis

PLS analysis was performed using SIMCA software (SIMCA-P 14.1, Umetrics Inc., Umea, Sweden) for regression analysis between the peak area of characteristic components and the antioxidant activities. In the PLS model, the areas of characteristic component peaks were the independent variables (X), and the scavenging activities of ABTS and DPPH were the dependent variables (Y).

## 4. Conclusions

In this study, the UHPLC-Q-Exactive Orbitrap MS with multivariate analysis approach was established to identify and compare the chemical profiles of rhizome and radix MSCP. A total of 91 compounds were tentatively identified, and 10 compounds were selected as quality control markers to distinguish them. Additionally, the ABTS and DPPH assays were used to evaluate their antioxidant efficiency. GRA and PLS analysis indicated that uvaol-3-caffeate (peak 91), 3-O-caffeoyloleanolic acid (peak 88), and khrinone E (peak 76) are the primary bioactive markers for their antioxidant activity. The radix of MSCP is a rich source to be considered as a natural antioxidant reagent. The study could be helpful for future exploration of its material base and bioactive mechanism. In addition, it would be considered to be used as a new method for the quality control of *M. speciosa*.

## Figures and Tables

**Table 1:** The mass spectrometry data and identification of rhizome and radix in MSCP.

NO.	Retention Time (min)	ESI-MS (*m*/*z*)	Error (ppm)	MS/MS Fragments Ions	Formula	Identification
1	3.31	315.0715 [M-H]^−^	−0.24	152.0101, 109.0202	C_13_H_16_O_9_	Protocatechuic acid-4-glucoside
2	3.57	315.0714 [M-H]^−^	−0.53	152.0101, 109.0201	C_13_H_16_O_9_	Protocatechuic acid-3-glucoside
3	4.44	421.1330 [M+H]^−^	0.16	289.0921, 128.0442, 127.0387, 97.0285, 85.0287	C_17_H_24_O_12_	NCGC00380493-01
4	4.54	461.1290 [M-H]^−^	0.39	329.0862, 167.0336, 152.0101, 108.0202	C_19_H_26_O_13_	Saccharumoside C
5	4.74	461.1292 [M-H]^−^	0.39	329.0858, 167.0336, 152.0101, 108.0202	C_19_H_26_O_13_	Saccharumoside D
6	4.86	315.0716 [M-H]^−^	−0.05	153.0179, 109.0280	C_13_H_16_O_9_	Protocatechuic acid-2-glucoside
7	5.13	447.1136 [M-H]^−^	0.56	315.0716, 152.0101, 108.0201	C_18_H_24_O_13_	5-{[2-O-(beta-d-apiofuranosyl)-beta-d-glucopyranosyl]ox y}-2-hydroxybenzoic acid
8	5.17	447.1137 [M-H]^−^	0.54	315.0715, 152.0102, 108.0202	C_18_H_24_O_13_	4-Hydroxy-5-(3′,4′,5′-Trihydroxyphenyl)-Valeric Acid-O-Methyl-O-Glucuronide
9	5.23	477.1603 [M-H]^−^	0.09	345.1177, 183.0651, 168.0414, 153.0179	C_20_H_30_O_13_	Shamiminol
10	6.00	188.0703 [M+H]^−^	−1.40	170.0594, 143.0728, 118.0652, 115.0542, 91.0545	C_11_H_9_O_2_N	trans-3-Indoleacrylic acid
11	6.06	247.1434 [M+H]^−^	−3.8	188.0701, 146.0598, 118.0650 60.0873,	C_14_H_18_O_2_N_2_	Hypaphorine
12	6.07	188.0704 [M+H]^−^	−1.4	170.0595, 143.0720, 118.0651, 115.0514, 91.0544	C_11_H_9_O_2_N	Indole-3-crylic acid
13	6.69	577.1352 [M-H]^−^	1.96	407.0760, 289.0711, 245.0816, 161.0233, 125.0299	C_30_H_26_O_12_	Procyanidin B2
14	6.88	477.1602 [M-H]^−^	0.09	345.1178, 183.0650, 168.0415, 153.0179	C_20_H_30_O_13_	Kelampayoside A
15	7.07	289.0708 [M-H]^−^	0.47	245.0809, 137.0229, 125.0229, 109.0280	C_15_H_14_O_6_	Epicatechin
16	7.18	581.1500 [M-H]^−^	−0.13	287.0554, 269.0449, 259.0605, 151.0023	C_26_H_30_O_15_	5,7,3′,4′-Tetrahydroxyflavanone-7-alpha-L-arabinofuranosyl-(1->6)-glucoside
17	7.32	449.1078 [M-H]^−^	0.29	287.0553, 269.0446, 259.0602, 174.4993, 151.0020	C_21_H_22_O_11_	Eriodictyol-7-O-glucoside
18	7.58	505.1703 [M-H]^−^	−0.35	343.1176, 325.1073, 310.0838,	C_25_H_30_O_11_	Diosbulbinoside D
19	7.70	563.1396 [M-H]^−^	0.10	431.0963, 269.0448	C_26_H_28_O_14_	Apiin
20	7.93	583.1662 [M-H]^−^	0.77	167.0361, 152.0103, 123.0432	C_26_H_32_O_15_	Seguinoside K
21	7.95	613.1751 [M-H]^−^	0.29	338.5223, 197.0443, 182.0204, 161.0439, 153.0543, 139.0382, 115.9812,	C_27_H_34_O_16_	Albibrissinoside B
22	8.02	431.1182 [M-H]^−^	−0.49	299.0775, 137.0320, 93.0330	C_18_H_24_O_12_	Apiosylglucosyl-4-hydroxybenzoate
23	9.09	271.0604 [M-H]^−^	1.81	243.0654, 227.0702, 225.0548, 163.0025, 135.0074, 109.0280, 91.0174	C_15_H_12_O_5_	3′,4′,7-Trihydroxyflavanone
24	9.12	445.1127 [M-H]^−^	−0.50	283.0605, 268.0370, 240.0420, 224.0464, 212.0472, 135.0073	C_22_H_22_O_10_	Calycosin-7-O-beta-D-glucoside
25	9.49	271.0611 [M-H]^−^	0.97	243.0654, 227.0731, 225.0540, 163.0028, 135.0075, 109.0280, 91.0173	C_15_H_12_O_5_	3′,4′,7-trihydroxyisoflavanone
26	9.97	627.1902 [M-H]^−^	−1.7	556.2740, 459.1497, 183.0654, 167.0336, 152.0102, 123.0437	C_28_H_36_O_16_	Khaephuoside B
27	10.26	271.0597 [M+H]^−^	−3.6	253.0482, 225.0542, 215.0690, 197.0593, 137.0230	C_15_H_10_O_5_	5,3′,4′-Trihydroxyflavone
28	10.37	137.0229 [M-H]^−^	−2.31	93.0329	C_7_H_6_O_3_	Salicylic acid
29	10.49	271.0606 [M-H]^−^	0.97	253.0505, 135.0437, 134.0358, 91.0173	C_15_H_12_O_5_	butein
30	10.64	187.0963 [M-H]^−^	−1.2	169.0850, 143.1064, 125.0956, 97.0643	C_9_H1_6_O_4_	Azelaic acid
31	10.71	577.1554 [M-H]^−^	0.37	445.1126, 283.0606	C_27_H_30_O_14_	Yuankanin
32	10.74	271.0601 [M-H]^−^	−0.31	243.0654, 227.0715, 163.0028, 135.0073, 109.0280, 91.0173	C_15_H_12_O_5_	2′,4′,7-trihydroxyisoflavanone
33	10.91	269.0448 [M-H]^−^	0.32	241.0495, 227.0430, 225.0524, 224.0544, 213.0547, 195.0441, 185.0593, 135.0073, 133.0280, 91.0173	C_15_H_10_O_5_	Sulfuretin
34	10.95	273.0758 [M-H]^−^	0.22	255.0658, 227.0699, 167.0336, 137.0299, 109.0280	C_15_H_14_O_5_	2′,4,4′,a-tetrahydroxy-dihydrochalcone
35	10.98	445.1131 [M-H]^−^	0.33	283.0605, 268.0369, 239.0343, 224.0472, 212.0464, 151.0032, 132.0202	C_22_H_22_O_10_	Sissotrin
36	11.68	285.0760 [M-H]^−^	0.27	270.0511, 269.0411, 253.0500, 241.0487, 270.0511, 253.0500, 241.0487, 180.0051, 161.0230, 148.0148, 135.0444, 123.0436, 91.0173	C_16_H_14_O_5_	Vestiton
37	11.82	285.0748 [M+H]^−^	0.92	270.0515, 242.0564, 225.0535, 197.0583, 137.0230	C_16_H_12_O_5_	5,4′-dihydroxy-3′-methoxy-isoflavone
38	11.91	253.0497 [M-H]^−^	−3.5	223.0395, 208.0521, 195.0440, 180.0568, 135.0072, 91.0173	C_15_H_11_O_4_	Daidzein
39	12.02	255.0659 [M-H]^−^	2.72	135.0075, 119.0487, 91.0174	C_15_H_12_O_4_	dihydrodaidzein
40	12.20	255.0652 [M-H]^−^	0.25	135.0074, 119.0487, 91.0173	C_15_H_12_O_4_	Liquiritigenin
41	12.31	283.0602 [M-H]^−^	0.38	268.0371, 251.0337, 239.0342, 224.0495, 211.0388, 195.0441, 183.0441, 167.0484, 156.0567, 148.0153, 135.0073, 91.0173	C_16_H_12_O_5_	Isoprunetin
42	12.40	285.0758 [M-H]^−^	−0.36	270.0520, 228.1277, 194.5045, 149.0230, 135.0073, 121.0283, 91.0173	C_16_H_14_O_5_	3′,4′-Dihydroxy-7-methoxyflavanone
43	12.62	315.0850 [M-H]^−^	2.12	176.0103, 163.0020, 135.0071	C_17_H_16_O_6_	Violanone
44	12.65	285.0758 [M-H]^−^	−0.38	270.0520, 269.0444, 228.1277, 194.5045, 149.0230, 135.0073, 121.0283, 91.0173	C_16_H_14_O_5_	3′,7-Dihydroxy-4′-methoxyisoflavanone
45	12.72	283.0603 [M-H]^−^	1.2	268.0370, 251.0348, 239.0339, 224.0465, 211.0387, 195.0440, 183.0445, 167.0490, 156.0565, 148.0152, 135.0073, 91.0173	C_16_H_12_O_5_	2′-Hydroxyformononetin
46	12.79	297.0398 [M-H]^−^	0.33	269.0488, 253.0501, 241.0490, 225.0548, 211.0396, 197.0596, 181.0643, 161.0228, 147.0069, 135.0075, 91.0173	C_16_H_10_O_6_	Griffonianone H
47	12.88	285.0759 [M-H]^−^	0.39	270.0520, 228.1277, 194.5045, 163.0365, 148.0190, 147.0433, 135.0073, 121.0283, 91.0173	C_16_H_14_O_5_	3,8-Dihydroxy-9-methoxypterocarpan
48	13.34	297.0762 [M-H]^−^	1.41	282.0527, 267.0291, 254.0577, 239.0340, 223.0391, 221.0392, 195.0439, 183.0437, 167.0488, 132.0199	C_17_H_14_O_5_	8-O-Methylretusin
49	13.38	269.0799 [M-H]^−^	1.2	254.0555, 226.0610, 151.0413, 118.0411	C_16_H_12_O_4_	4′-hydroxy-7-methoxyisoflavone
50	13.51	269.0812 [M-H]^−^	0.38	253.0499, 237.0547, 225.0549, 211.0394, 161.0225, 145.0278, 133.0188, 117.0331, 108.0201	C_16_H_14_O_4_	medicarpin
51	13.58	255.0655 [M-H]^−^	1.2	135.0073, 119.0487	C_15_H_12_O_4_	5,4′-dihydroxy-flavanone
52	13.83	301.0699 [M+H]^−^	−2.40	286.0463, 269.0443, 241.0485, 229.0488, 153.0177, 134.0351	C_16_H_12_O_6_	2′-Hydroxybiochanin A
53	14.15	271.0598 [M-H]^−^	−1.81	253.0488, 135.0436, 134.0359, 91.0173	C_15_H_12_O_5_	Garbanzol
54	14.18	297.0759 [M-H]^−^	0.66	282.0526, 267.0292, 254.0577, 239.0341, 223.0390, 221.0389, 195.0438, 183.0439, 167.0488, 132.0202	C_17_H_14_O_5_	Afrormosin
55	14.35	271.0606 [M-H]^−^	0.36	187.0385, 165.0176, 151.0022, 119.0487, 107.0123	C_15_H_12_O_5_	Naringenin
56	14.40	287.0912 [M+H]^−^	−0.52	269.0803, 254.0559, 226.0622, 198.0659, 153.0544, 135.0437, 107.0492	C_16_H_14_O_5_	6,7-Dihydroxy-4′-methoxyisoflavanone
57	14.40	271.0957 [M+H]^−^	−2.54	151.0386, 119.0489	C_16_H_14_O_4_	2′,4-dihydroxy-4′-methoxychalcone
58	14.55	297.0761 [M-H]^−^	0.65	282.0526, 267.0293, 254.0575, 239.0340, 223.0392, 221.0390, 195.0437, 183.0439, 167.0489, 132.0202	C_17_H_14_O_5_	Alfalone
59	14.66	301.0701 [M+H]^−^	−0.59	286.0467, 285.0336, 269.0647, 241.0486, 229.0486, 213.0538, 184.0517, 139.0541, 93.0377	C_16_H_12_O_6_	Tectorigenin
60	14.67	283.0604 [M-H]^−^	1.02	268.0370, 239.0334, 224.0468, 211.0387, 195.0443, 183.0436, 167.0491, 135.0073, 91.0174	C_16_H_12_O_5_	Calycosin
61	14.85	285.0747 [M+H]^−^	−3.75	267.0634, 239.0691, 211.0750, 196.0515, 151.0386	C_16_H_12_O_5_	3′,4′-Dihydroxy-7-methoxyisoflavone
62	14.88	301.0697 [M+H]^−^	1.35	286.0456, 285.0329, 269.0433, 241.0488, 229.0487, 213.0532, 187.0383, 153.0177, 134.0359	C_16_H_12_O_6_	Pratensein
63	15.01	315.0850 [M+H]^−^	−4.08	300.0615, 272.0644, 257.0429, 255.0637, 244.0714, 240.0440, 227.0696, 216.0788, 212.0449, 201.0551, 175.0338, 167.0335, 152.0096, 148.0514, 133.0228,	C_17_H_14_O_6_	5,4′-Dihydroxy-7,3′-dimethoxyisoflavone
64	15.12	315.0504 [M-H]^−^	2.4	300.0644, 284.0270, 256.0344, 148.0128, 125.0229	C_16_H_12_O_7_	3′,5,6,7-Tetrahydroxy-4′-methoxyisoflavone
65	15.15	283.0604 [M-H]^−^	1.13	268.0369, 256.0370, 239.0344, 224.0467, 211.0388, 195.0444, 183.0441, 167.0489, 132.0201	C_16_H_12_O_5_	Glycitein
66	15.25	285.0761 [M-H]^−^	1.36	270.0526, 267.0656, 255.0288, 241.0493, 224.0466, 211.0395, 183.0438, 153.0699, 149.0230	C_16_H_14_O_5_	3′,4′-Dihydroxy-7-methoxyisoflavanone
67	15.32	313.0346 [M-H]^−^	0.33	285.0393, 269.1279, 257.0460, 245.0443, 227.0341, 217.0496, 203.0340, 175.0387, 161.0231, 151.0022, 149.0231, 133.0282, 109.0277, 107.0123,	C_16_H_10_O_7_	Luteolal
68	15.51	301.0712 [M-H]^−^	0.43	286.0425, 151, 0028, 125.0229	C_16_H_14_O_6_	Ferreirin
69	15.55	281.0446 [M-H]^−^	0.92	253.0496, 223.0398, 208.0524, 195.0441, 180.0564, 167.0487, 155.0476, 135.0070, 132.0200, 91.0173	C_16_H_10_O_5_	Pseudobaptigenin
70	15.60	257.0802 [M+H]^−^	−3.5	137.0229, 119.0491, 93.0368	C_15_H_12_O_4_	Isoliquiritigenin
71	15.68	329.2324 [M-H]^−^	0.78	229.1436, 211.1329, 183.1377, 171.1014	C_18_H_34_O_5_	9,12,13-Trihydroxy-10-octadecenoic acid
72	15.75	267.0656 [M-H]^−^	1.2	252.0418, 223.0390, 208.0522, 195.0439, 180.0563, 167.0487, 135.0071, 132.0202, 91.0174	C_16_H_12_O_4_	Formononetin
73	15.88	297.0758 [M-H]^−^	−2.5	284.0671, 269.0441, 256.0725, 241.0491, 282.0527, 267.0291, 254.0576, 239.0342, 223.0391, 221.0389, 195.0438, 183.0440, 167.0496, 132.0202	C_17_H_14_O_5_	7-O-Methylbiochanin A
74	15.93	297.0757 [M-H]^−^	−0.34	282.0525, 267.0291, 254.0577, 239.0340, 223.0402, 221.0386, 195.0451, 183.0445, 167.0497, 135.0202	C_17_H_14_O_5_	Cladrin
75	15.96	297.0396 [M-H]^−^	2.3	269.0446, 241.0494, 225.0542, 213.0548, 197.0594, 183.0440, 161.0225, 147.0073, 133.0327	C_16_H_10_O_6_	5,7-dihydroxy-3′,4′-methylenedioxyisoflavone
76	16.15	313.0710 [M-H]^−^	1.00	298.046, 297.0396, 283.0215, 269.0449, 254.0580, 225.0544, 161.0299, 149.0277, 135.0071, 121.0208, 91.0173	C_17_H_14_O_6_	Khrinone E
77	16.28	285.0745 [M+H]^−^	−1.92	270.0910, 255.0642, 163.0388, 151.0385, 147.0432, 123.0438, 93.0336	C_16_H_12_O_5_	Homopterocarpin
78	16.69	271.0968 [M+H]^−^	0.34	147.0433, 137.0593, 123.0438	C_16_H_14_O_4_	Echinatin
79	16.78	941.5099	1.02	923.4996, 879.5104, 795.4318, 633.4003, 615.3887, 597.3769, 553.3872, 457.3671, 437.3403, 409.3475, 247.0819, 205.0713, 163.0610, 157.0147, 143.0339, 139.0029	C_48_H_78_O_18_	Soyasaponin I
80	16.95	329.0657 [M-H]^−^	0.46	314.0406, 313.0349, 286.0451, 285.0392, 271.0222, 245.0480, 177.0179, 152.0100, 151.0025, 136.0102, 107.0122	C_17_H_14_O_7_	Tricin
81	17.19	283.0604 [M-H]^−^	0.81	269.0403, 268.0367, 239.0341, 223.0390, 195.0451, 183.0441, 167.0491, 135.0074, 132.0202, 91.0174	C_16_H_12_O_5_	maackiain
82	17.22	315.0867 [M-H]^−^	1.32	300.0629, 285.0401, 241.0492, 214.9861, 196.0004, 164.0103, 151.0023, 107.0122	C_17_H_16_O_6_	Homoferreirin
83	19.72	295.2271 [M-H]^−^	1.0	277.2159, 171.1024	C_18_H_32_O_3_	9-hydroxyoctadeca-10,12-dienoic acid
84	21.58	271.2273 [M+HCOO]^−^	1.19	226.2241, 225.2216	C_15_H_30_O	2-Pentadecanone
85	21.71	457.3676 [M+H]^−^	0.36	439.3570, 381.3133, 248.1692, 191.1788	C_30_H_48_O_3_	betulinic acid
86	22.04	617.3844 [M-H]^−^	1.02	453.3359, 163.0392, 145.0273, 119.0495	C_39_H_54_O_6_	27-p-Coumaroyloxyursolic acid
87	22.06	617.3851 [M-H]^−^	1.6	437.3422, 179.0339, 161.0243, 134.0365	C_39_H_54_O_6_	3-β-O-trans-caffeoylbetulinic acid
88	22.08	617.3853 [M-H]^−^	2.3	437.3417, 179.0345, 161.02441, 134.0363	C_39_H_54_O_6_	3-O-Caffeoyloleanolic acid
89	22.38	339.2321 [M-H]^−^	0.3	163.1115	C_23_H_32_O_2_	2,2′-Methylenebis
90	22.47	603.4050 [M-H]^−^	1.5	179.0341, 161.0240, 134.0367	C_39_H_56_O_5_	Betulin-3-caffeate
91	22.51	603.4051 [M-H]^−^	0.6	179.0333, 161.0240, 134.0364	C_39_H_56_O_5_	uvaol-3-caffeate

**Table 2:** Sample collection information and antioxidant activity of MSCP (*n* = 3).

No.	Collecting Location	GPS Data	Collection Year	Classification	ABTS	DPPH
					IC_50_(μg/mL)	IC_50_(μg/mL)
A1	Pubei, Guangxi	N22°15′12″ E 109°34′25″	June. 2019	radix	3.10 ± 0.13	5.16 ± 0.49
A2	Pubei, Guangxi	N22°0′33″ E 109°28′47″	June. 2019	radix	3.94 ± 0.18	5.41 ± 0.32
A3	Hexian, Guangxi	N22°41′57″ E 109°19′38″	June. 2019	radix	4.56 ± 0.28	5.25 ± 0.11
A4	Shangxi, Guangxi	N22°09′43.45″ E 108°08′35″	Oct. 2019	radix	3.57 ± 0,16	4.83 ± 0.79
A5	Shangxi, Guangxi	N22°07′30″ E 108°06′54″	Oct. 2019	radix	2.09 ± 0.13	2.76 ± 0.21
A6	Jiangmen, Guangdong	N22°34′30″ E 113°02′45″	May. 2020	radix	3.64 ± 0.35	4.84 ± 0.73
A7	Jiangmen, Guangdong	N22°34′43″ E 113°02′41″	May. 2020	radix	4.34 ± 0.32	5.77 ± 0.15
A8	Heshan, Guangdong	N22°42′35″ E 113°1′16″	May. 2020	radix	3.73 ± 0.18	4.89 ± 0.98
A9	Heshan, Guangdong	N22°42′35″ E 113°0′16″	May. 2020	radix	3.59 ± 0.26	4.11 ± 0.42
A10	Taizhou, Zhejiang	N28°38′17″ E 121°16′25″	June. 2020	radix	3.51 ± 0.14	4.21 ± 0.88
A11	Taizhou, Zhejiang	N28°38′28″ E 121°16′20″	June. 2020	radix	3.99 ± 0.20	4.48 ± 0.76
A12	Nanning, Guangxi	N22°30′7″ E 108°08′35	June. 2020	radix	4.15 ± 0.65	4.85 ± 0.48
A13	Nanning, Guangxi	N22°23′54″ E 108°27′43″	Aug. 2020	radix	3.10 ± 0.18	5.11 ± 0.67
A14	Nanning, Guangxi	N22°24′38″ E 108°29′23″	Aug. 2020	radix	3.45 ± 0.51	4.20 ± 0.36
A15	Qinzhou, Guangxi	N22°25′4.50″ E 109°35′18″	Aug. 2020	radix	3.26 ± 0.14	5.47 ± 0.22
A16	Qinzhou, Guangxi	N22°26′35″ E 109°41′19″	Aug. 2020	radix	3.61 ± 0.22	5.66 ± 0.55
B1	Pubei, Guangxi	N22°15′12″ E 109°34′25″	June. 2019	rhizome	7.39 ± 0.54	7.81 ± 0.65
B2	Pubei, Guangxi	N22°0′33″ E 109°28′47″	June. 2019	rhizome	6.15 ± 0.16	6.46 ± 0.74
B3	Hexian, Guangxi	N22°41′57″ E 109°19′38″	June. 2019	rhizome	6.45 ± 0.29	7.72 ± 1.93
B4	Shangxi, Guangxi	N22°09′43″ E 108°08′35″	Oct. 2019	rhizome	5.19 ± 0.34	6.3 ± 0.63
B5	Shangxi, Guangxi	N22°07′30″ E 108°06′54″	Oct. 2019	rhizome	9.01 ± 1.24	10.14 ± 2.2
B6	Jiangmen, Guangdong	N22°34′30″ E 113°02′45″	May. 2020	rhizome	5.78 ± 0.33	7.40 ± 0.79
B7	Jiangmen, Guangdong	N22°34′43″ E 113°02′41″	May. 2020	rhizome	6.00 ± 0.21	8.12 ± 0.99
B8	Heshan, Guangdong	N22°42′35″ E 113°1′16″	June. 2020	rhizome	7.91 ± 0.22	9.69 ± 0.44
B9	Heshan, Guangdong	N22°42′357″ E 113°0′16″	June. 2020	rhizome	5.64 ± 0.33	7.06 ± 0.79
B10	Taizhou, Zhejiang	N28°38′17″ E 121°16′25″	Aug. 2020	rhizome	5.05 ± 0.26	5.86 ± 0.48
B11	Taizhou, Zhejiang	N28°38′28″ E 121°16′20″	Aug. 2020	rhizome	10.13 ± 1.20	11.92 ± 0.75
B12	Nanning, Guangxi	N22°30′7″ E 108°08′35″	Aug. 2020	rhizome	5.33 ± 0.24	7.21 ± 0.18

**Table 3:** Correlations and grade of GRA.

Peaks	ABTS		DPPH	
	Correlations	Rank	Correlations	Rank
a	0.7070	6	0.6778	7
b	0.6412	9	0.7087	4
c	0.7846	1	0.7811	1
d	0.7407	3	0.7016	5
e	0.7750	2	0.7770	2
f	0.7114	4	0.7433	3
g	0.6893	8	0.6344	8
h	0.6901	7	0.6841	6
i	0.7073	5	0.5975	10
j	0.5651	10	0.6098	9

## Data Availability

Not applicable.
